# Modification of Leaf Glucosinolate Contents in *Brassica oleracea* by Divergent Selection and Effect on Expression of Genes Controlling Glucosinolate Pathway

**DOI:** 10.3389/fpls.2016.01012

**Published:** 2016-07-15

**Authors:** Tamara Sotelo, Pablo Velasco, Pilar Soengas, Víctor M. Rodríguez, María E. Cartea

**Affiliations:** Group of Genetics, Breeding and Biochemistry of Brassicas, Misión Biológica de Galicia-Consejo Superior de Investigaciones CientíficasPontevedra, Spain

**Keywords:** divergent mass selection, glucosinolates, *Brassica oleracea*, GSL-ALK, RT-qPCR, modifying gene expression

## Abstract

Modification of the content of secondary metabolites opens the possibility of obtaining vegetables enriched in these compounds related to plant defense and human health. We report the first results of a divergent selection for glucosinolate (GSL) content of the three major GSL in leaves: sinigrin (SIN), glucoiberin (GIB), and glucobrassicin (GBS) in order to develop six kale genotypes (*Brassica oleracea* var. *acephala*) with high (HSIN, HIGIB, HGBS) and low (LSIN, LGIB, LGBS) content. The aims were to determine if the three divergent selections were successful in leaves, how each divergent selection affected the content of the same GSLs in flower buds and seeds and to determine which genes would be involved in the modification of the content of the three GSL studied. The content of SIN and GIB after three cycles of divergent selection increased 52.5% and 77.68%, and decreased 51.9% and 45.33%, respectively. The divergent selection for GBS content was only successful and significant for decreasing the concentration, with a reduction of 39.04%. Mass selection is an efficient way of modifying the concentration of individual GSLs. Divergent selections realized in leaves had a side effect in the GSL contents of flower buds and seeds due to the novo synthesis in these organs and/or translocation from leaves. The results obtained suggest that modification in the SIN and GIB concentration by selection is related to the GSL-ALK locus. We suggest that this locus could be related with the indirect response found in the GBS concentration. Meantime, variations in the CYP81F2 gene expression could be the responsible of the variations in GBS content. The genotypes obtained in this study can be used as valuable materials for undertaking basic studies about the biological effects of the major GSLs present in kales.

## Introduction

Glucosinolates (GSLs) are a major class of secondary metabolites found in the family *Brassicaceae*. Due to their enhanced plant protection to biotic and abiotic stresses (Fahey et al., 2001; Santolamazza–Carbone et al., 2014) and their preventive effects on several human cancers (Fahey and Stephenson, 1999; Forte et al., 2008), they have been extensively investigated. The hydrolytic breakdown products of GSLs, especially isothiocyanates (ITCs), have beneficial effects on human health, such as cytotoxic and apoptotic effects in damaged cells, preventing cancer in humans and reducing risk for degenerative diseases (Cartea and Velasco, 2008; Forte et al., 2008; Van Horn et al., 2008; Virgili and Marino, 2008). In contrast, in rapeseed meal, the dominant GSL, progoitrin (2-hidroxy-3-butenyl GSL, PRO) is changed into an oxazolidine-2-thione, which causes goiter and has other detrimental effects on animal health (Liu et al., 2012).

GSLs are sulfur-rich plant secondary metabolites with a basic skeleton consisting of a β-thioglucose residue, an N-hydroxy monosulfate moiety, and a variable side chain (Halkier and Du, 1997; Kliebenstein et al., 2001b). Generally, GSLs are divided into three different classes according to the amino acid precursor in biosynthesis and are called aromatic GSLs (derived from phenylalanine or tyrosine), aliphatic GSLs (derived from methionine, alanine, valine, leucine, and isoleucine) and indolic GSLs (synthesized from tryptophan) (Zukalova and Vasak, 2002; Bekaert et al., 2012).

GSL biosynthesis is a tripartite pathway involving three independent steps: (i) side chain elongation, which is carried out by methylthioalkylmalate synthase enzymes (MAM). (ii) Development of the core structure, which includes several steps: aldoxime formation catalyzed by the CYP79 family of cytochromes P450; aldoxime oxidation by the CYP83 family; thiohydroximic acid formation by conjugation to an S donor and after C-S bond cleavage; desulfoGSL formation by S-glucosyltransferase (S-GT); and GSL formation by sulfotransferase. (iii) Secondary modification of the amino acid side chain, which includes oxidation, hydroxylation, methoxylation, desaturation, sulfation and glycosylation (Sorensen, 1988; Mikkelsen et al., 2002). Side chain elongation and development of the core structure are common to the three types of GSLs biosynthesis (Figure S1).

It is known that three loci mainly determine the profile and content of aliphatic GSLs in *B. oleracea*. The presence of 3C-GSL is controlled by a dominant allele of GSL-PRO whereas the presence of 4C-GSL and 5C-GSL is controlled by a dominant allele of GSL-ELONG. Another major gene involved in the synthesis of aliphatic GSL is GSL-ALK, which controls the conversion of methylsulphinyl GSL into alkenyl GSL (Li et al., 2001), a step related with the production of sinigrin (2-propenyl, SIN) and gluconapin (3-butenyl, GNA). The indolic GSLs pathway has been less studied than the aliphatic GSLs one. There are key loci that synthesized the core structure of indolic GSLs biosynthesis such as CYP79B2, CYP79B3, or CYP83B1 (Mikkelsen et al., 2000; Bak et al., 2001; Naur et al., 2003).

The increase of beneficial GSLs and the reduction of detrimental GSLs are a target in brassica improvement in order to obtain crops with high value and improved food quality. On the other hand, the obtaining of plant material with the same genetic background but with different concentrations of specific GSLs will allow us to study their biological effects. The first modification of GSLs content by classical breeding took place in the 70s, when low erucic acid and low GSLs content varieties of *B. napus* were obtained by introgression from other *B. napus* cultivars (Stefansson and Kondra, 1975; Röbbelen and Thies, 1980). In the 90s, UK groups held a screening of diverse wild *Brassica* species and found that *Brassica villosa* contained a high concentration of glucoraphanin (4-methylsulphinylbutyl, GRA). This wild species was crossed with a commercial broccoli leading to the production of a new cultivar of broccoli enriched in GRA (Mithen et al., 2003; Sarikamis et al., 2006). More recently, molecular biology techniques were applied to modify the content of a particular GSL. Liu et al. (2012) obtained *B. napus* seeds enriched in GRA though the GSL-ALK silencing using RNAi.

The accumulation and profile of GSLs are highly dependent on the genotype, although they are also affected by environmental and developmental factors (Kliebenstein et al., 2001a; Brown et al., 2003). The concentration of GSLs shows a high variability among species, different varieties of the same species or even among plants of the same variety (Kushad et al., 1999). Divergent mass selection has been widely used in plant breeding as it can generate groups of individuals that share the same genetic background but with extreme values for a particular trait. Stowe and Marquis (2011) used this type of selection to effectively modify the total GSLs content of leaves of a rapid cycling variety of *B. rapa*. This kind of selection could also be used to modify the content of a particular GSL. The content and profile of these secondary metabolites vary with plant organs (Brown et al., 2003; Velasco et al., 2007). Selection carried out in one organ could produce side effects on the content of GSLs in other organs of the plant. Modification or selection by one gene of the GSLs biosynthetic pathway can also produce alterations or modifications in the concentration of other GSLs within the same biosynthetic pathway. On the other hand, it would be interesting to know the action of which genes are being modified in the process of divergent selection. This information will allow us to select directly for those genes that are involved in this change.

In kales (*Brassica oleracea* var. *acephala*), two aliphatic GSLs, SIN and glucoiberin (3-methylsulphinylpropyl, GIB), and one indolic GSL, glucobrassicin (3-indolylmethyl, GBS), are the predominant in the leaf profile (Velasco et al., 2007; Cartea and Velasco, 2008). We report herein the results of three cycles of divergent mass selection for GIB, SIN and GBS content in leaves. This on-going selection program provides unique germplasm to study the direct and indirect effects of selection on individual GSLs concentration. Our objectives were: (1) studying the effect to the divergent selections for the content of two aliphatic GSLs (GIB and SIN) and one indolic GSL (GBS) in leaves, (2) determining the side effect of divergent selections in seeds and flower buds, (3) establishing whether the content of other GSLs may be altered with the selections carried on in leaves and (4) determining which genes would be involved in the modification of the leaf content of GIB, SIN, and GBS in divergent selections.

## Materials and methods

### Divergent selection program

Divergent selections were started in 2006 by using seeds of the kale population MBG-BRS0062, kept at the brassica germplasm bank at Misión Biológica de Galicia (MBG-CSIC) (Galicia, NW Spain). The population presents variability for GSL concentration and this is a desirable characteristic to realize a mass divergent selection for high and low content. Divergent selections were designed to obtain plant varieties with high (HSIN) and low (LSIN) SIN content, high (HGIB) and low (LGIB) GIB content, and high (HGBS) and low (LGBS) GBS content. In 2006, approximately 750 plants from cycle 0 (C0) were transplanted in the field into six cages (125 plants each for each one of the selections, i.e., HSIN, LSIN, HGIB, LGIB, HGBS, and LGBS. The leaf GSL content of all the plants was assessed 120 days after sowing by UHPLC. After analysis, the 25 plants (≈20% selection intensity) with the highest content were selected in HSIN, HGIB and HGBS, and the 25 plants with the lowest content were selected in LSIN, LGIB and LGBS. Non-selected plants were removed from the cages before flowering. Cross-pollination among the selected plants in each cage was made by bumblebees (*Bombus terrestris*). Afterwards, seed of the selected plants were mixed and in this way the cycle 1 (C1) of each one of the selections was obtained. From 2008 to 2009, this process was repeated to obtain the cycles C2 and C3, respectively for all the high and low selections. After finishing the process of selection, and in order to recombine each one of the genotypes and to obtain seed for each selection cycles in the same environmental conditions, all the selection cycles plus the original population (C0, C1, C2, and C3 for each SIN, GIB, and GBS) were multiplied in 2010 in isolated experimental plots at MBG-CSIC.

### Evaluation trials

Two different assays were carried out. A field trial was conducted to test the effectiveness of divergent selection. Recombined plants from 18 cycles of divergent selection (C1, C2, and C3 for HSIN, LSIN, HGIB, LGIB, HGBS, and LGBS) plus the original cycle (C0) were studied in the same year in order to avoid variations on GSLs content due to environmental conditions. The study was conducted during 2012 at MBG-CSIC (Galicia, NW Spain). Plants were grown in multi-pot trays under controlled conditions in an acclimatized greenhouse from July to August in 2012. On 29th August plants were transplanted into the field (Salcedo, NW Spain, 42° 24′N, 8° 38′W) at 5–6 true leaf stages. Experimental design was a randomized complete block with three replicates. Each plot had two rows spaced 0.8 m and each row consisted of 15 plants spaced 0.6 m.

The evaluation of C0 with the same precision than the other cycles requires a considerably larger number of experimental plots, as this population contained 100% of the initial variability for GSL concentration. For this reason, three plots of the C0 were planted per block, while for the other genotypes one plot per block was planted. This variability was of less magnitude in the rest of cycles, because their starting variability had been reduced by the first cycle of selection. Cultivation operations, fertilization, and weed control were carried out according to local practices and crop requirements. Leaf samples were harvested on ≈90 days old plants. The third leaf of a total of 20 healthy and competitive plants from each plot was chosen as plant material for GSLs analysis. Leaf samples were divided in two different bulks. Flower bud samples were collected from the same experimental plot sequentially, depending on the flowering time of each variety. In this case, 15 flower buds were collected and divided in three bulks from each plot. Tissue samples from leaves and flower buds were stored at −80°C, freeze-dried and ground until GSLs analysis. Five 100 mg bulks of the recombined seeds obtained in 2011 for each genotype were ground and analyzed to study the GSLs profile and GSLs content. Different agronomic traits were evaluated in the divergent selections according previous studies in this crop (Padilla et al., 2007; Vilar et al., 2008). These traits were: early vigor by using a subjective scale from 1 (very poor) to 5 (excellent); late vigor by using a subjective scale from 1 (very poor) to 5 (excellent); leaf fresh matter as the average fresh weight of a leaf (g) (mean of 25 leaves per plot taken from 5 plants per plot); leaf moisture as the percentage of fresh weight of a fresh leaf (%) and time to flowering as the number of days from transplanting until 50% of plants have the first flower.

A second assay under controlled conditions was conducted with recombined plants from the C3 (high and low) of each divergent selection and C0 in order to relate the GSL content of the plants and the expression of the principal genes related with their biosynthesis. Plants were grown in multi-pot trays in a growth chamber at 25°C ± 2°C for days and 20°C ± 2°C at night. Plants were harvested 3 months after germination and stored at −80°C until use. Three biological replicates with approximately 35 plants each one, were collected by cycle and then, each replicate were divided into three bulks. These bulks were employed to study GSL content and gene expression.

### GSL identification and quantification

GSL extraction was conducted on samples of both trials. In field assay, GSLs were analyzed in leaves, flower buds and seeds, while in the assay under controlled conditions GSLs were analyzed in leaves. Sample extraction and desulfation, were performed according to Kliebenstein et al. (2001b) with minor modifications. Two microliters of the desulfo-GSL extract for seeds and flower buds and three microliters for leaves were used to identify and quantify the GSLs. The chromatographic analyses were carried out on an Ultra-High-Performance Liquid-Chromatograph (UHPLC Nexera LC-30AD; Shimadzu) equipped with a Nexera SIL-30AC injector and one SPD-M20A UV/VIS photodiode array detector. The UHPLC column was a C18 Atlantis® T3 waters column (3 μm particle size, 2.1 × 100 mm i.d.) protected with a C18 guard cartridge. The oven temperature was set at 30°C. Compounds were separated using the following method in aqueous acetonitrile, with a flow of 0.8 mL min^−1^: 1.5 min at 100% H_2_O, an 11 min gradient from 0% to 25% (v/v) acetonitrile, 1.5 min at 25% (v/v) acetonitrile, a minute gradient from 25% to 0% (v/v) acetonitrile, and a final 3 min at 100% H_2_O. Data was recorded on a computer with the LabSolutions software (Shimadzu). All GSLs (the three major ones under selection and other minor GSLs) were quantified at 229 nm by using SIN (sinigrin, monohydrate from Phytoplan, Diehm & Neuberger GmbH, Heidelberg, Germany) and GBS (glucobrassicin, potassium salt monohydrate, from Phytoplan, Diehm & Neuberger GmbH, Heidelberg, Germany) as external standard and expressed in μmol g^−1^ dry weight (DW). Calibration equations were made with, at least, five data points, from 0.34 to 1.7 nmol for SIN and from 0.28 to 1.4 nmol for GBS. The average regression equations for SIN, and GBS were *y* = 148.818 × (*R*^2^ = 0.99), *y* = 263.822 × (*R*^2^ = 0.99), respectively.

### Total RNA extraction, primer design, and cDNA synthesis

Leaf RNA from three biological replicates of C0 and the C3 of each divergent selection HSINC3, LSINC3, HGIBC3, LGIBC3, HGBSC3, and LGBSC3, was isolated from 100 mg of ground samples using a Spectrum™ Plant Total RNA Kit (Quiagen, Valencia, CA, USA). Total RNA concentration was quantified using NanoDrop 1000 (Thermo Scientific, Waltham, MA, USA). To remove any traces of genomic DNA from extractions, the RNA was treated with RQ1 RNase-Free DNase (Promega, CA, USA) following the manufacturer's instructions. The cDNA was synthesized from 1 μg of total RNA using a GoScript™ Reverse Transcription System, according to the manufacturer's instructions (Promega, Madison, WI, USA).

Quantitative reverse transcription-PCR (RT-qPCR) was employed to analyze the expression patterns of 12 genes including a housekeeping gene namely glyceraldehyde-3-phosphate- dehydrogenase (GADPH) and the following genes related to the aliphatic GSLs pathway:UDP-glycosyltransferase 74B1 (UGT74B1), desulfoglucosinolate sulfotransferase (St5a), S-alkyl-thiohydroximate lyase (SUR1), glutathione S-transferase PHI 10 (GSTF10), γ-glutamyl peptidase 1 (GGP1), transcription factor (MYB51), Cytochrome P450 monooxygenase (CYP81F2) and 2-oxoglutarate-dependent dioxygenase (ALK). Finally, several genes related to the indolic and aromatic GSLs pathways were also studied: transcription factor (ATR1), cytochrome P450 83B1 (CYP83B1), Tryptophan N-monooxygenase 1(CYP79B2) and Tryptophan N-monooxygenase 2 (CYP79B3) were the aromatic and indolic genes studied.The RT-qPCR primers were designed from previously identified sequences of the GLS biosynthetic route obtained in the website http://plants.ensembl.org. Primers were designed at http://bioinfo.ut.ee/primer3-0.4.0 and they are shown in Table S1.

In order to determine specificity of primers designed in the current study, agarose gel electrophoresis and melting curve analyses were performed. All the primer pairs amplified single PCR products of expected size (Table S1) and the specificity of amplicon was confirmed by the presence of single peak during melt curve. RT-qPCR was performed using a Promega kit in a total volume of 15 μl. After denaturation at 95°C for 10 min, 40 cycles were performed under the following conditions: 95°C for 15 s and 60°C for 60 s. Primer efficiency was calculated using the LingRegPCR software (Ramakers et al., 2003) and results were normalized to GADPH expression. RT-qPCRs were carried out on a 7500 Real Time PCR System (Applied Biosystem, Forster City, CA, USA).

### Statistical analysis

Combined analyses of variance across selection cycles for total and individual GSLs, agronomical traits and relative gene expression were computed using the PROC GLM of SAS v 9.2 program (SAS, 2011). Population means were compared using the Fisher protected Least Significant Difference test (LSD, *p* ≤ 0.05). Besides, simple linear regression analyses were performed for the GSL implied in the three divergent selections (SIN, GIB, and GBS) as dependent variables and cycles of selection as independent variables for each organ under study (leaves, flower buds, and seeds).

Simple linear regression analyses where the GSLs under selection were the independent variables and the other GSLs, the sum of aliphatic, indolic and total GSLs were the dependent variables were also performed. Correlation coefficients between gene expression and GSLs concentration and between expressions of the different genes were computed with PROC CORR of SAS program v 9.2 (SAS, 2011).

## Results

### Direct response to divergent selection for sinigrin, glucoiberin, and glucobrassicin content in leaves and associated response in agronomical traits

**Figure 1:**
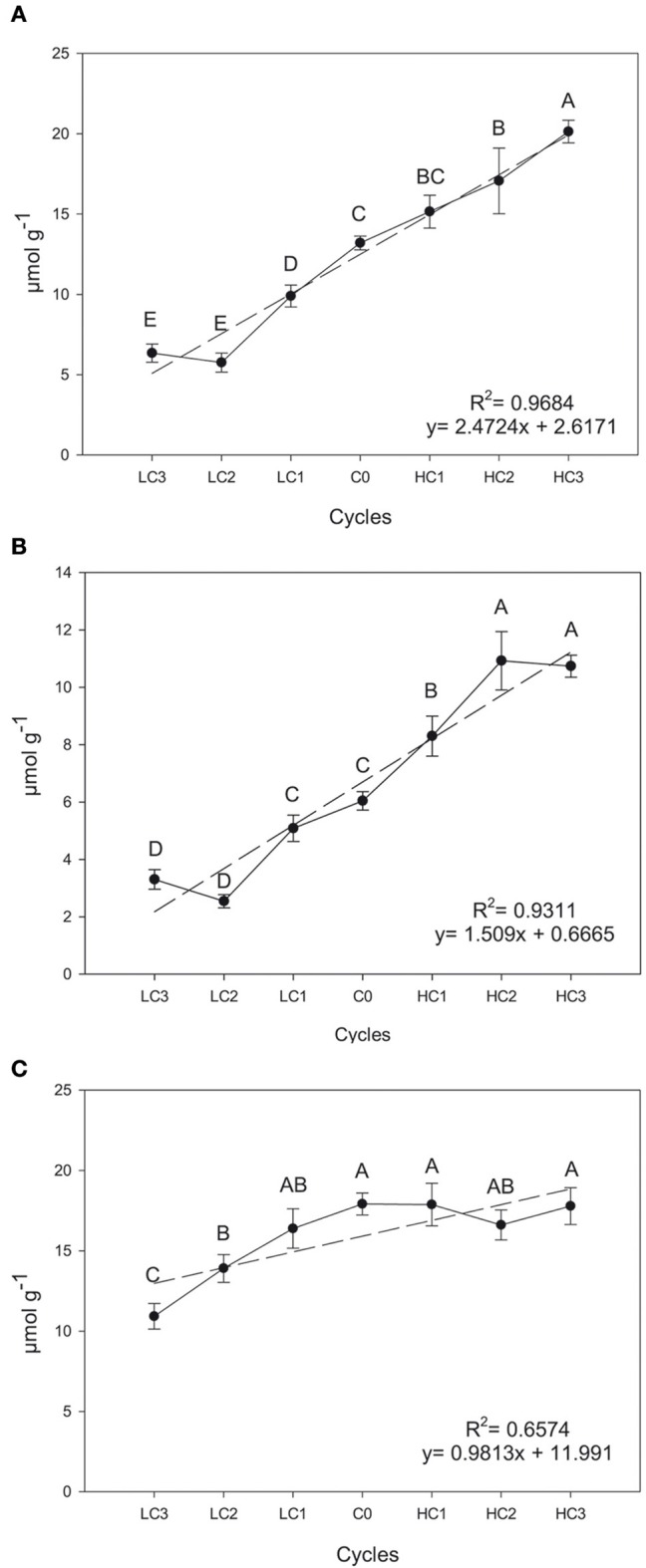
**Graphical representations of simple linear regression divergent selection in leaves for the content (μmol g^**−1**^) for sinigrin (A), glucoiberin (B) and glucobrassicin (C)**. Data are means of three biological replicates and error bars are ± *P* < 0.05. LC1, low cycle 1; LC2, low cycle 2; LC3, low cycle 3; C0, original cycle; HC1, high cycle 1; HC2, high cycle 2; HC3, high cycle 3.

Significant and positive simple linear regression coefficients across selection cycles for SIN (*R*^2^= 0.9684, *P* ≤ 0.0001), GIB (*R*^2^ = 0.9311, *P* = 0.0004) and GBS (*R*^2^ = 0.6574, *P* ≤ 0.0001) concentration were observed in leaves (Figure 1). Generally speaking, the response to divergent selection for the three GSLs was effective and linear in leaves; therefore, mass selection is an efficient way of increasing or decreasing the concentration of individual GSLs.

The content of SIN and GIB after three cycles of divergent selection increased 52.5% (*P* = 0.0074) and 77.68% (*P* = 0.0410), respectively, and decreased 51.9% (*P* = 0.0322) and 45.33% (*P* = 0.0385), respectively. Meantime, the divergent selection performed for the leaf GBS content, was only successful and significant for decreasing the concentration, with a reduction of 39.04% (*P* = 0.0248).

Analysis of variance showed that there were not significant differences for any agronomic trait across divergent selections (data not shown).

### Response to divergent selection for sinigrin, glucoiberin, and glucobrassicin in other organs

There were significant and positive linear regressions between the SIN concentrations in leaves and the concentration of SIN in flower buds and seeds across selection. The same response was obtained in the other GSLs under selection, GIB and GBS although values of the *R*^2^ for GBS were low (Table 1). Therefore, selection performed in leaves had a side effect in flower buds and seeds.

**Table 1:** **Coefficients for simple linear regressions where sinigrin, glucoiberin, and glucobrassicin in leaves are the independent variables and the other GSLs present in leaves, flower buds and seeds are the dependent variables**.

		**SIN**			**GIB**			**GBS**		
		**Leaves**	**Flower buds**	**Seeds**	**Leaves**	**Flower buds**	**Seeds**	**Leaves**	**Flower buds**	**Seeds**
GIB	*R*^2^	0.2072	0.0065	0.7698		0.7102	0.5055	0.0124	0.0405	0.0876
	a	−2.636	−0.2499	−0.838^**^		0.6513^**^	0.1758^**^	0.1230	0.1767	−0.0920
SIN	*R*^2^		0.5511	0.3986	0.8022	0.5466	0.3050	0.2699	0.1627	0.0187
	a		0.7688^**^	0.1413^**^	−1.044^**^	−0.189^**^	−0.078^**^	1.0200	0.5091	−0.0176
GBS	*R*^2^	0.0001	0.1770	0.0037	0.8621	0.1396	0.0808		0.2873	0.2687
	a	0.0167	0.7173^*^	−2.7113	0.639^**^	0.3307	−3.4549		0.2171^**^	7.527^**^
PRO	*R*^2^	0.5642	0.0358	0.0223	0.1589	0.0191	0.6797	0.0871	0.0473	0.0580
	a	12.325^*^	1.5229	0.2861^*^	−5.552	−0.4538	−0.773^**^	3.3980	2.5197	0.0774
GRA	*R*^2^	–	0.1082	0.3042	–	0.4500	0.4772	–	0.0452	0.0294
	a	–	−8.6995	−5.9581	–	6.6729^**^	5.0037^**^	–	2.0638	1.2440
GNA	*R*^2^	0.0928	–	0.0614	0.4870	–	0.551	0.0040	–	0.0416
	a	−19.22	–	1.4190^*^	−28.623	–	−1.723^**^	3.6650	–	0.1844
OHGBS	*R*^2^	0.4880	0.0151	0.1161	0.0167	0.0205	0.0433	0.7135	0.2969	0.0141
	a	32.822	−3.4938	0.9019	7.937	0.3094	−0.3005	0.4861^**^	−8.2618^**^	0.1726
NeoGBS	*R*^2^	0.0923	0.0207	0.0021	0.5209	0.0040	0	0.9331	0.6181	0.1600
	a	−4.632	0.4645	−2.9566	4.004	0.1439	0.0295	3.202^**^	1.0279^**^	7.5880
GNT	*R*^2^	0.2049	0.0030	0.0084	0.0035	0.0299	0.4134	0.1026	0.1974	0.0556
	a	−7.821	−1.5807	−2.1207	0.773	−2.5277	−8.888^**^	−4.7210	3.3897^*^	−2.5483
Aliphatics	*R*^2^	0.9735	0.1245	0.0203	0.1863	0.0013	0.0060	0.1442	0.0191	0.0002
	a	1.101^**^	0.1571	0.02234	1.000	−0.0085	−0.0061	0.368	0.0292	0.0010
Indolics	*R*^2^	0	0.0517	0.0168	0.8599	0.0041	0.0067	0.9961	0.1270	0.0291
	a	−0.008	0.1279	0.2802	0.581^**^	0.0166	−0.0797	0.762^**^	0.0853	0.1857
Total	*R*^2^	0.7630	0.0875	0.0201	0.9105	0.0001	0.0065	0.7341	0.0614	0.0014
	a	0.8171^*^	0.0725	0.0208	0.545^**^	0.0011	−0.0058	0.438^**^	0.0279	0.0025

*Aliphatic glucosinolates: GIB, Glucoiberin; SIN, Sinigrin; GRA, Glucoraphanin; GNA, Gluconapin; PRO, Progoitrin; Indolic glucosinolates: OHGBS, 4-hydroxyglucobrassicin; GBS, Glucobrassicin; NeoGBS, Neoglucobrassicin: Aromatic glucosinolate: GNT, Gluconasturtiin. R^2^: coefficient of determination of each glucosinolate. a: slope of the line*.

* *Significant at P ≤ 0.05*,

and

** *significant at P ≤ 0.01*.

**Figure 2:**
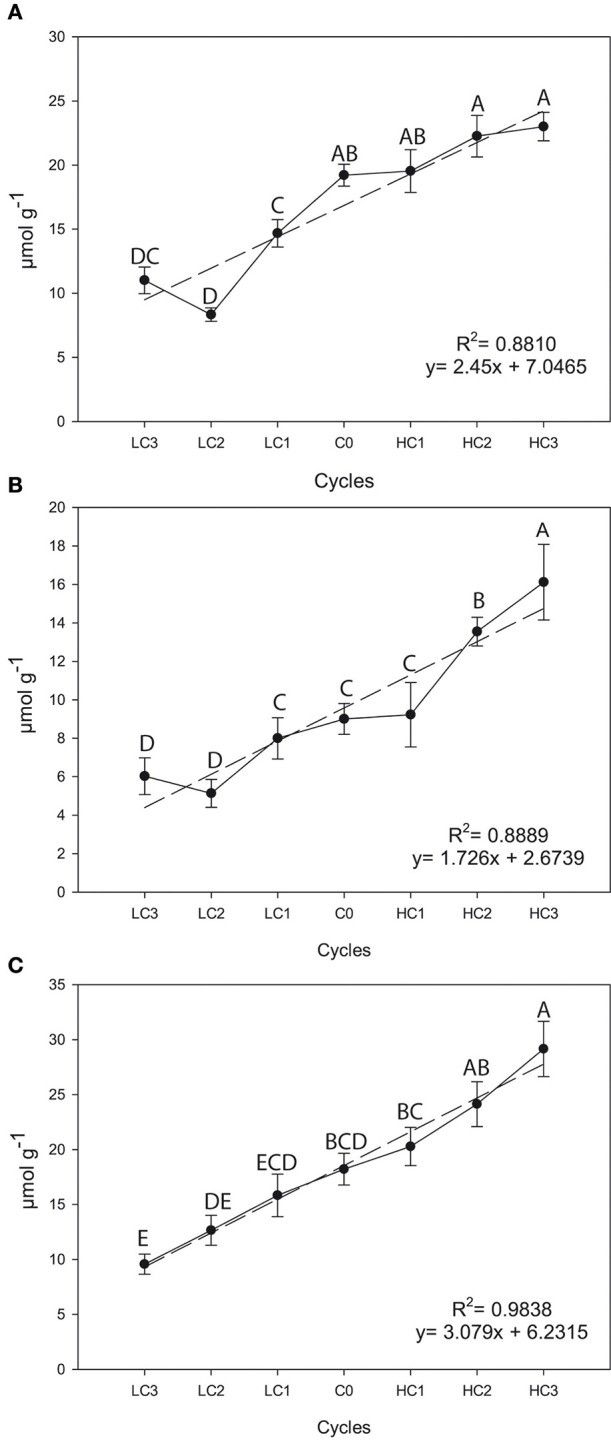
**Graphical representations of simple linear regression divergent selection in flower buds for the content (μmol g^**−1**^) for sinigrin (A), glucoiberin (B) and glucobrassicin (C)**. Data are means of three biological replicates and error bars are ± *P* < 0.05. LC1, low cycle 1; LC2, low cycle 2; LC3, low cycle 3; C0, original cycle; HC1, high cycle 1; HC2, high cycle 2; HC3, high cycle 3.

There were significant differences among selection cycles for the three GSLs in flower buds. Significant and positive simple linear regression coefficients for SIN (*R*^2^ = 0.8810, *P* = 0.0017), GIB (*R*^2^ = 0.8889, *P* = 0.0015) and GBS (*R*^2^ = 0.9838, *P* ≤ 0.0001) across selection cycles were found (Figure 2). There was a 19.7% (*P* = 0.0511) increase in SIN, a 79.62% (*P* = 0.0461) increase in GIB and a 60.02% (*P* = 0.0160) increase in GBS after three selection cycles vs. the original cycle. Meantime, the decrease in the content for SIN was 42.73% (*P* = 0.0153), 33.05% (*P* = 0.0142) for GIB and 47.60% (*P* = 0.0010) for GBS.

**Figure 3:**
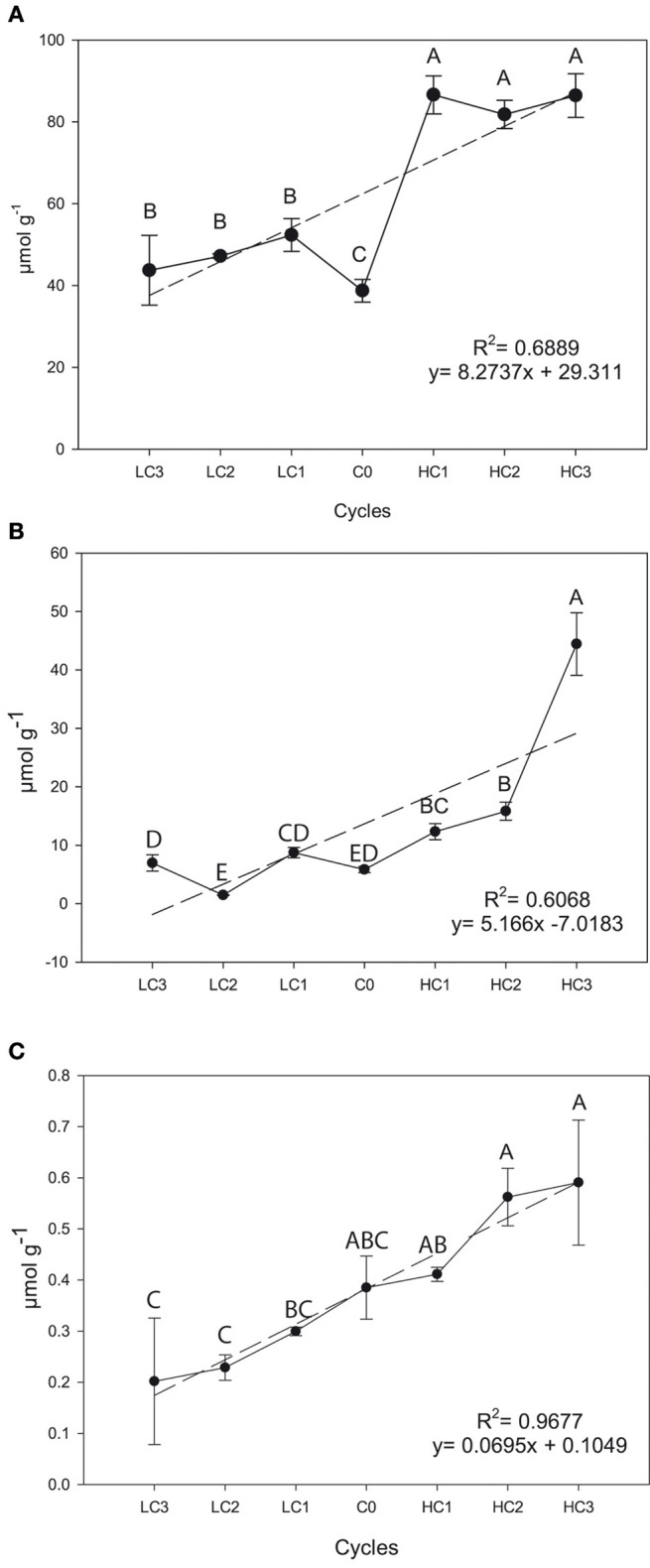
**Graphical representations of simple linear regression divergent selection in seeds for the content (μ6mol g^**−1**^) for sinigrin (A), glucoiberin (B) and glucobrassicin (C)**. Data are means of three biological replicates, and error bars are ± *P* < 0.05. LC1, low cycle 1; LC2, low cycle 2; LC3, low cycle 3; C0, original cycle; HC1, high cycle 1; HC2, high cycle 2; HC3, high cycle 3.

Positive and simple linear regressions were also found for SIN (*R*^2^ = 0.6889 *P* = 0.0208), GIB (*R*^2^ = 0.6068, *P* = 0.0390) and GBS (*R*^2^ = 0.9677, *P* = 0.0010) in seeds (Figure 3). For aliphatic GSLs, selection was successful to increase the SIN and GIB concentration but selection was unsuccessful for GBS. The increase was 123.23% (*P* = 0.012) in SIN, and 661.78% (*P* ≤ 0.001) in GIB and 53.35% (*P* = 0.0584) in GBS, meantime GBS was reduced in a 47.58% (*P* = 0.0532) although there are no significant differences.

### Indirect response to divergent selection on other GSLs and relationship to gene expression

Besides the three major GSLs under selection, this population also presents other GSLs as the aliphatics progoitrin (PRO), glucoraphanin (GRA) and gluconapin (3- butenyl, GNA), the aromatic gluconasturtiin (2-phenethyl, GNT) and the indolics, hidroxyglucobrassicin (4-hydroxy-3-indolylmethyl, OHGBS) and neoglucobrassicin (1-methoxy-3-indolylmethyl, NEOGBS) (Table 2). A regression analysis was made with the leaf SIN, GBS, and GIB content as independent variables and the content of the other GSLs in leaves, flower buds and seeds as dependent variables (Table 1). Significant and positive regressions were found between the leaf SIN content across selection cycles and PRO, aliphatic GSLs and total GSLs in leaves, GBS in flower buds and GNA in seeds. A negative correlation coefficient was found for GIB in seeds. By modifying the content of SIN, a positive related response was found in the content of PRO and GNA and a negative response in the content of GIB.

**Table 2:** **Glucosinolate (GSL) concentration (μmol g^**−1**^ dw) of the original cycle (C0) of the kale population for the three organs under study**.

**Glucosinolate**	**Leaves^b^**	**Flower buds**	**Seeds**
GIB^a^	6.045	9.002	5.831
SIN^a^	13.202	19.219	38.724
GBS^a^	17.920	18.221	0.385
PRO	1.032	1.046	8.212
GRA	0.000	0.507	0.578
GNA	0.040	0.000	2.112
OHGBS	0.250	0.418	2.127
NeoGBS	2.698	4.178	0.292
GNT	1.717	1.261	0.305
Aliphatics	20.319	29.774	56.016
Indolics	20.868	22.816	2.804
TOTAL	42.905	53.852	59.125

a *Glucosinolates studied in the three divergent selections*.

b *Organ where selection was performed. Aliphatic glucosinolates: GIB, Glucoiberin; SIN, Sinigrin; GRA, Glucoraphanin; GNA, Gluconapin; PRO, Progoitrin; Indolic glucosinolates: OHGBS, 4-hydroxyglucobrassicin; GBS, Glucobrassicin; NeoGBS, Neoglucobrassicin; Aromatic glucosinolate: GNT, Gluconasturtiin*.

In the divergent selection program for leaf GIB content, significant and positive regressions were found between leaf GIB content and SIN and GBS, total indolic GSLs and total GSLs in leaves and GRA in flower buds and seeds (Table 1). Negative relationships were found between the leaf GIB content and PRO, GNA, and GNT in seeds and SIN in both seeds and flower buds.

**Figure 4:**
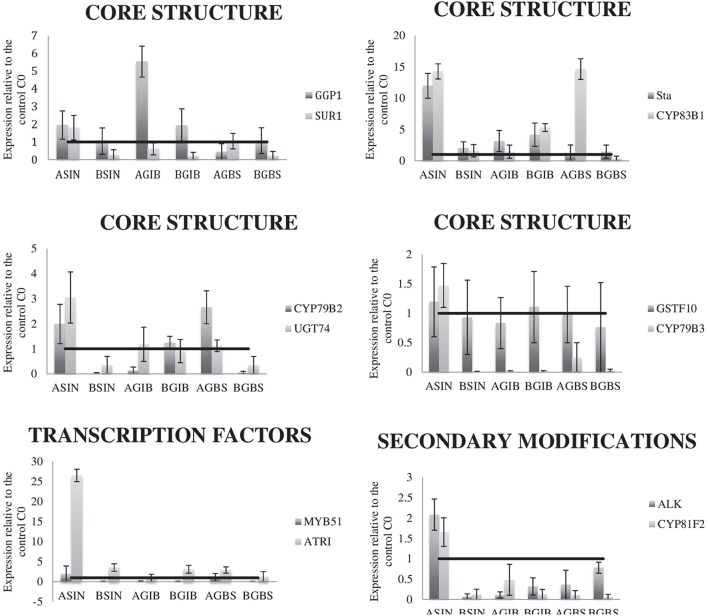
**Graphical representation of gene expression levels in the C3 of the three divergent selections relative to the expression of the same genes in the control (C0)**. Data are means of three biological replicates, and the error bars indicate their standard deviations. The horizontal line in each graph indicates the relative expression of each gene in the control. LC1, low cycle 1; LC2, low cycle 2; LC3, low cycle 3; C0, original cycle; HC1, high cycle 1; HC2, high cycle 2; HC3, high cycle 3.

An assay was performed to relate variation in the GSLs content in the cycles 3 (C3) of each selection with the relative expression of several genes related to their biosynthesis. The expression of 12 genes, related to the core biosynthesis of GSLs, to secondary modifications and to transcription factors were studied. The expression levels of all the genes are higher in C3 of HSIN than in C0 (Figure 4). Meantime in the C3 of LSIN the expression of SUR1, GSTF10, CYP79B3, CYP79B2, UGT74, MYB51, ALK, and CYP81F2 decreased respect to C0 (Figure 4). However, analysis of variance showed that no one of these differences are significant (data not shown), probably due to the high variability found among replicates of RT-qPCR. Relative gene expression was only significant for GGP1 in GIB selection, which expression is higher in HGIB and LGIB than in C0. However, regression analysis showed significant associations between gene expression and GSLs content across selection cycles.

High and significant correlations between SIN and GIB concentration and GSL-ALK expression were found (Table 3, Figure 4). When SIN concentration increases, GSL-ALK gene expression also increases (*r* = 0.92); however, when GIB concentration increases, the expression of GSL-ALK gene decreases (*r* = −0.82). Variation in SIN concentration causes significant and positive correlations with the majority of the other genes studied while variation in GIB only causes a correlated and negative response in CYP79B3 (Table 3, Figure 4). In the case of SIN selection, expression of GSL-ALK gene presents a positive and significant correlation with CYP79B2 (*r* = 0.967), CYP79B3 (*r* = 0.991), CYP83B1 (*r* = 0.958), SUR1 (*r* = 0.958), UGT74B1 (*r* = 0.971) and St5a (*r* = 0.942). Meantime, in the case of GIB divergent selection, GSL-ALK expression showed significant and positive correlations with CYP79B2 (*r* = 0.780) and CYP83B1 (*r* = 0.966) genes.

**Table 3:** **Significant correlations between Sinigrin (SIN), Glucoiberin (GIB) and Glucobrassicin (GBS) concentration and expression of 12 genes related to the glucosinolate biosynthetic route**.

		**Core structure**								**Secondary modifications**	
		**CYP79B2**	**CYP79B3**	**CYP83B1**	**GSTF10**	**GGP1**	**SUR1**	**UGT74B1**	**St5a**	**CYP81F2**	**ALK**
SIN	Correlation	0.8356	0.9377	0.9538	0.0116	−0.5030	0.8171	0.8712	0.8837	0.8389	0.9206
	*P*-value	0.0103	0.0015	0.0012	0.1052	0.0770	0.0083	0.0076	0.0008	0.0057	0.0012
GIB	Correlation	−0.2179	−0.9475	−0.2699	0.0381	0.6680	0.5567	−0.4150	0.3552	0.2622	−0.8276
	*P*-value	0.0678	0.0001	0.0756	0.0666	0.0599	0.0601	0.0764	0.1222	0.1323	0.0033
GBS	Correlation	−0.1829	−0.1159	0.2985	0.1302	0.0249	0.4752	0.8485	0.4935	0.7879	−0.5388
	*P*-value	0.1211	0.0644	0.0652	0.0989	0.0657	0.0654	0.0019	0.0696	0.0431	0.0635

In the divergent selection for the leaf GBS content, significant and positive regression was found with the content of OHGBS, NEOGBS, total indolic GSLs and total GSLs (Table 1). GBS is the precursor of OHGBS and NeoGBS in the biosynthetic pathway of indolic GSLs (Figure S2B); therefore, variation in GBS content provokes a positive response in the leaf content of NeoGBS and OHGBS.

As in the case of aliphatic selections, there were no significant differences in gene expression across selection cycles. However, significant correlations of gene expression with GBS content were found. The expression of CYP83B1 was positively correlated to GBS content (Figure 4). This gene is responsible of the conversion of GBS into OHGBS.

A significant regression of GBS with the aromatic GSL GNT was found in flower buds although the R^2^ was low. Indolic and aromatic GSLs share the gene UGT74B1 in their pathways (Figure S2B), which expression was modified with the content of GBS (Figure 4).

The relationship of the expression of the indolic regulators, ATRI and MYB51 genes with the content of GBS was also studied, but they were no significant.

## Discussion

### Direct response to divergent selection for sinigrin, glucoiberin, and glucobrassicin content in leaves and associated response in agronomical traits

After three cycles of divergent selection, the response to divergent selection for the three GSLs under study was effective and linear in kale leaves. The effect of selecting for GSLs content did not have any effect in agronomical traits; therefore, mass selection is an efficient way of increasing or decreasing the concentration of individual GSLs. A modification in the concentration of the aliphatic GSLs (SIN and GIB) was observed in both senses of the divergent selections. Stowe and Marquis (2011) obtained similar results in a divergent selection to modify the content of total GSLs in *B. rapa*. However, the divergent selection performed for the leaf GBS content was only successful and significant for decreasing the concentration. The asymmetric response in a divergent selection program, such as we found for GBS content, has been found before, for example in maize for leaf chlorophyll content but the cause is still unknown (Korkovelos and Goulas, 2011). There are some possible causes to explain this effect such as differential selection, genetic asymmetry, selection for heterozygotes, inbreeding depression or maternal effects (Falconer, 1989).

The mass selection is an effective method for highly heritable traits. Although the estimates of heritability could not be calculated with the experimental design used in our work, according to the results obtained, we can conclude that heritability should be high enough. In this sense, Madsen et al. (2014) in *B. napus* and Márquez-Lema et al. (2009) in *B. carinata*, estimated the heritability for total GSLs in seeds with values of *h*^2^ = 0.90 and *h*^2^ = 0.58, respectively. In another study, Van Doorn et al. (1998) established the heritability for two aliphatic GSLs (SIN and PRO) in different cultivars of Brussels sprouts with values of *h*^2^ = 0.77 and *h*^2^ = 0.79, respectively.

### Response to divergent selection for sinigrin, glucoiberin, and glucobrassicin in other organs

Leaves are the organ most consumed in kales, hence the importance to perform the divergent selections for specific GSLs in this organ. It has long been known that also there are GSLs in other organs such as roots, shoots, stems or seeds (Grubb and Abel, 2006) in part by the new GSLs biosynthesis or by translocation from other organs. We hypothesized that GSLs content on other organs, such as flower buds and seeds, could be affected by the selections performed in leaves.

When selection is carried out to increase the content of the three GSLs in leaves, there is also an increase of the same GSLs in flower buds and seeds except for SIN in flower buds and GBS in seeds. When the selection is carried out for decreasing the content of the three GSLs in leaves, there is also a reduction of the same GSLs in flower buds and no related responses were found in seeds for both aliphatic and indolic GSLs. The reproductive organs, including seeds, flowers and fruits, which contribute most to plant fitness, are expected to have the highest concentrations of GSLs. In this way, Brown et al. (2003) in *A. thaliana* demonstrate that seeds present higher content of GSLs than vegetative organs. GSLs accumulation represents the net effect of biosynthesis, transport and catabolism. It can be possible that, by modifying the action of genes responsible for the concentration of GSLs in leaves, the action of the same genes were also modified in flower buds and seeds.

Differences in concentration and pattern of GSLs in different organs of *B. rapa* were related to differential expression of transcription factors involved in GSLs biosynthesis (Clarke, 2010). In our case, the same response was found in leaves, flower buds and seeds; therefore, genes related to biosynthetic pathway and no transcription factors could be implied in the divergent selection. Besides, there is a translocation of GSLs from vegetative organs to reproductive ones with the development. Du and Halkier (1998) observed that the high accumulation of GSLs in seeds is not connected with a corresponding high level of associated biosynthesis, suggesting the involvement of transport processes. Chen et al. (2001) demonstrated the translocation of radiolabeled p-hydroxybenzyl GSL from leaves to seeds via phloem, either exogenously applied or de novo synthesized. In fact, a recent study in *A. thaliana* shows the necessary presence of one specific transporter for the GSL translocation from other organs to seeds (Nour-Eldin et al., 2012) and the necessary presence of these transporters related with the movement of GSLs from roots to shoots (Madsen et al., 2014).

### Indirect response to divergent selection on other GSLs and relationship to gene expression

The kale population studied in this work presents other GSLs as the aliphatics PRO, GRA, and GNA, the aromatic GNT and the indolic GSL, OHGBS, and NEOGBS which content could have been modified indirectly by the divergent selection performed on leaves for SIN, GIB, and GBS.

In the divergent selection for SIN, a positive correlation was found with the content of PRO and GNA and a negative correlation with the content of GIB suggesting that modification in the SIN concentration by selection is related to the GSL-ALK locus. GSL profile in Brassicaceae can be partially explained by genetic variation in the GSL-ALK locus encoding (2-oxoglutarate-dependent dioxygenase) which catalyzes the conversion of methylsulfinylalkyl GSL to the alkenyl form in plants (Li and Quiros, 2003). In the biosynthetic pathway of GSLs, the locus GSL-ALK controls the side chain desaturation and its presence determines the production of the alkenyl GSLs SIN (3C-GSL) and PRO and GNA (4C-GSL) (Li et al., 2001) (Figure S2A).

In the divergent selection program for leaf GIB content, there is a negative correlation of leaf content of GIB on the content of SIN, PRO and GNA, and a positive correlation on the content of GRA, which suggests that the modification of the content of GIB is related to the major locus, GSL-ALK. In the biosynthetic pathway of aliphatic 3C-GSLs, the alkenization of GIB produces SIN. In the pathway of 4C-GSLs, the alkenization of GRA produces GNA, which is afterwards transformed into PRO. Alkenizations are carried out by the GSL-ALK locus.

Supporting the role of GSL-ALK in modifying the content of SIN and GIB, high and significant correlations between SIN and GIB concentration and GSL-ALK expression were found. When SIN concentration increases, GSL-ALK gene expression also increases; however, when GIB concentration increases, the expression of GSL-ALK gene decreases. These results showed that different alleles of the GSL-ALK might be implied in these selections. The expression of GSL-ALK is correlated with the expression of as the genes CYP79B2, CYP79B3, CYP83B1, SUR1, UGT74B1, and St5a, all of them related to the synthesis of the core structure of aliphatic GSLs.

Recent evidence suggests a potential for feedback regulation in the GSL pathway. Genetic variation at GSL-ALK locus is linked to the production of alkenyl GSLs, but also to increase of total aliphatic GSL in *A. thaliana* (Kliebenstein et al., 2001a; Wentzell et al., 2007). Expression of the homologous of GSL-ALK (AOP2) from *B. oleracea* in a naturally occurring knockout genotype of *A. thaliana*, lead to the accumulation of alkenyl GSLs, doubling of total aliphatic GSL content and the induction of aliphatic GSL biosynthetic and regulatory genes. Wentzell et al. (2007) proposed that GSL-ALK has a regulatory effect in other genes of GSL synthesis trough a mechanism that is still unknown. More recently, Sotelo et al. (2014) found that GSL-ALK plays a central role in a network of epistatic interactions between ten QTLs related to GSLs, suggesting a possible regulatory effect of this locus in the GSL pathway.

By modifying the content of SIN, a positive response is also found for GBS and total indolic GSLs. Sotelo et al. (2014) found that GSL-ALK controls indirectly the variability for GBS content by epistatic interactions, indicating a cross talk between indolic and aliphatic pathways.

In the divergent selection for the leaf GBS content, results showed a significant and positive relationship with the content of OHGBS, NEOGBS, total indolic GSLs and total GSLs. GBS is the precursor of OHGBS and NeoGBS in the biosynthetic pathway of indolic GSLs (Figure S2B); therefore, variation in GBS content provokes a positive response in the leaf content of NeoGBS and OHGBS. In this case, we only found significant coefficients in leaves and flower buds, probably because the GBS levels in seeds are too low. Confirming these results, GBS content was correlated with CYP81F2 gene expression (Table 3), which catalyzes the conversion of GBS to OHGBS (Pfalz et al., 2009).

The relationship between GBS content and GSL GNT was only detected in flower buds, probably due to the higher concentration of GNT in flower buds than in leaves or seeds. Ours results suggest that expression UGT74B1 gene that is involved in the indolic and aromatic GSLs pathway (Figure S2B) was modified in relation with the content of GBS.

## Conclusions

Divergent mass selection for SIN, GIB, and GBS leaf content was successful indicating that there is high genetic variability within the population which allows us to modify the concentration of GSLs through mass selection. The genotypes obtained in this study (with increased and decreased GSL content) can represent valuable materials for undertaking basic studies about the biological effects of the major GSLs present in kales.

There was a side effect of divergent selection performed in leaves in the GSL content of flower buds and seeds, indicating modification of the synthesis of GSLs in these organs or translocation of GSLs from leaves. A further study to examine GSL-related gene expression changes, particularly GSL-ALK, in seeds and flower buds would be necessary to conclude if the changes of GSL contents in leaves during selection were caused by the reallocation of GSLs among different tissues/organs within plant or changes of GSL-related gene expression in leaves or both. Because kale plants have long vegetative periods (they are biannual), large heights, and it is very difficult to grow them in culture chambers to obtain flower buds, a new experiment in the field would be required in order to collect the buds and the seeds of each divergent selection and to perform further gene expression analyses.

Indirect effects of divergent selection performed for the two aliphatic GLS under selection (SIN and GIB) in the content of other GSLs suggest that different alleles of the locus GSL-ALK are responsible for the variation across the selection cycles. The expression of genes involved in the GSLs pathway confirmed these results. At the same time, this locus could be responsible of the indirect response found for the indolic GBS. In the case of indolic divergent selection, CYP81F2 gene could be the responsible of the variations in concentration across the selection cycles.

## Author contributions

TS carried out the experiments and wrote the manuscript. TS, PS, and VR performed the genetic analysis. TS and PV performed the glucosinolate analysis. PV, MC, and PS conceived the study and participated in its design. MC and PV coordinated the work. All authors have read and approved the manuscript.

## Funding

This work was supported by the National Plan for Research and Development AGL-2012-35539, AGL2015-66256-C2-1-R and financed by the European Regional Development Funds (FEDER).

### Conflict of interest statement

The authors declare that the research was conducted in the absence of any commercial or financial relationships that could be construed as a potential conflict of interest.

## Supplementary material

Figure S1 **Genetic model for the two first steps of the glucosinolates synthesis**. Extracted and modified of Redovnikovic et al. (2008).

Figure S2 **A biochemical genetic model of the biosynthesis of aliphatic glucosinolates (A) and indolic glucosinolates (B) in *Brassicaceae* including the major genes controlling this process**.

Table S1 **Primer sequences used for RT-qPCR and gene expression analysis**.
