# Supplementary figures and images for: Modification of Leaf Glucosinolate Contents in Brassica oleracea by Divergent Selection and Effect on Expression of Genes Controlling Glucosinolate Pathway
Source: Front Plant Sci. 2016 Jul 15;7:1012. doi: 10.3389/fpls.2016.01012 (PMC4945695; doi:10.3389/fpls.2016.01012)

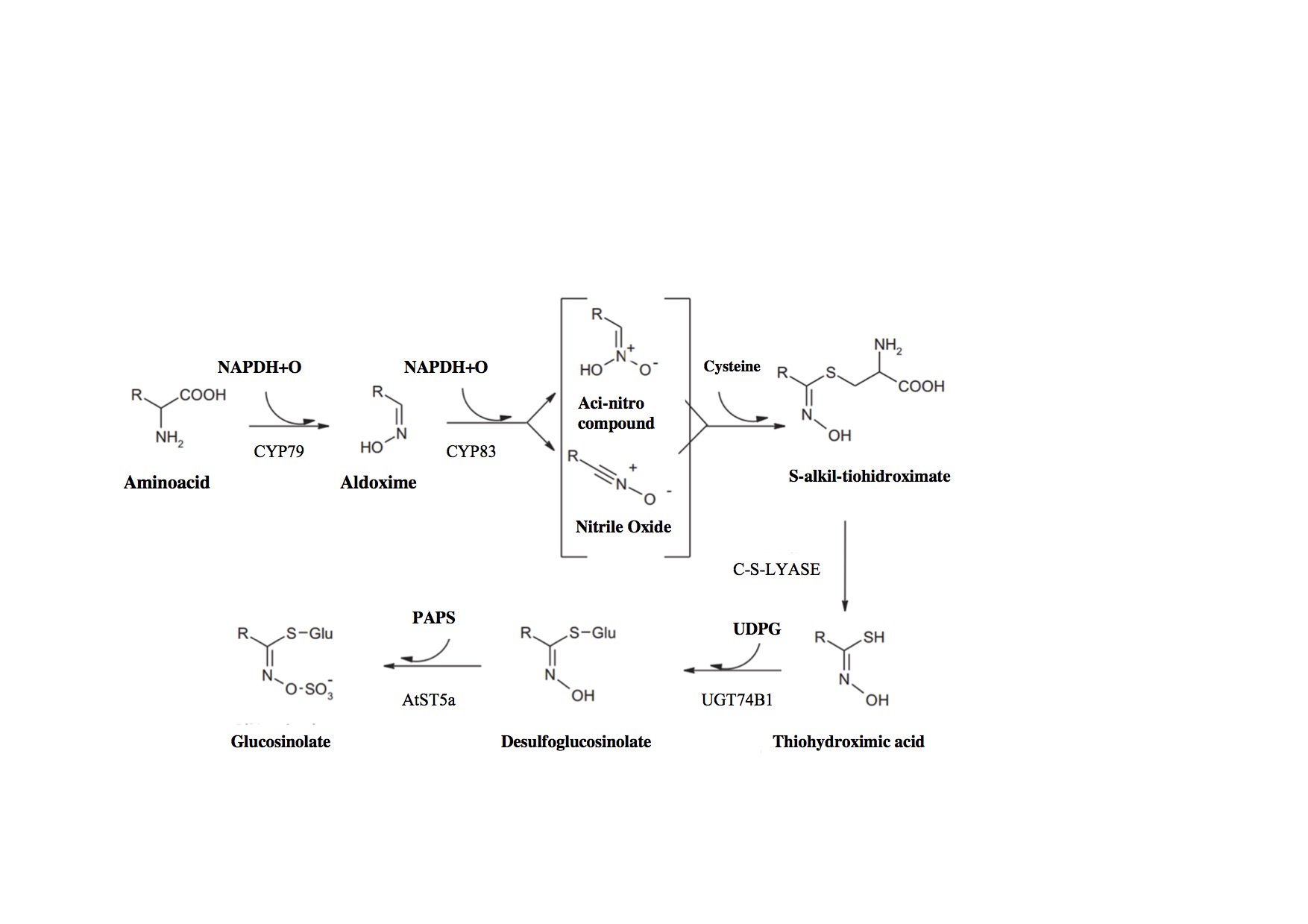

Supplement: Figure S1 — Genetic model for the two first steps of the glucosinolates synthesis. Extracted and modified of Redovnikovic et al. (2008). [file Image1.JPEG]

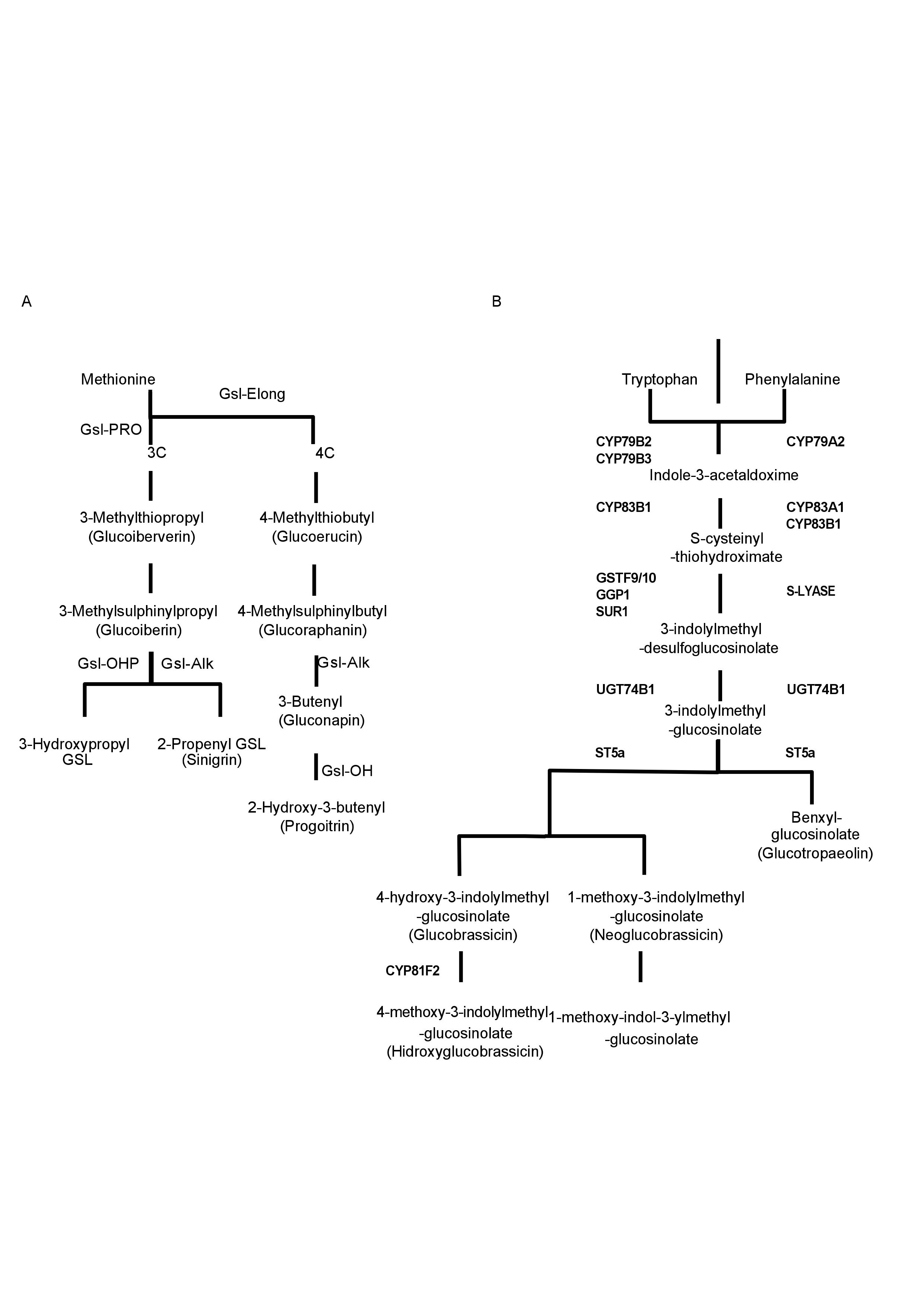

Supplement: Figure S2 — A biochemical genetic model of the biosynthesis of aliphatic glucosinolates (A) and indolic glucosinolates (B) in Brassicaceae including the major genes controlling this process. [file Image2.JPEG]
