# Supplementary material for: Modification of Leaf Glucosinolate Contents in Brassica oleracea by Divergent Selection and Effect on Expression of Genes Controlling Glucosinolate Pathway
Source: Front Plant Sci. 2016 Jul 15;7:1012. doi: 10.3389/fpls.2016.01012 (PMC4945695; doi:10.3389/fpls.2016.01012)
Supplement: Table S1 — Primer sequences used for RT-qPCR and gene expression analysis. [file Table1.PDF]

Table S1: Quantitative RT-PCR primer sequences used for gene expression analysis

Gen name		Sequence (5’→3’)	Part of biosynthetic route	Product length
**UGT74B1**	Forward primer<br>Reverse primer	ACGATAAAGGCTACGGCTCG<br>CCTCAGCGAGTTGCTTCTCA	Core structure	141
**St5a**	Forward primer<br>Reverse primer	ATCCCAAACGGGTCGTTACC<br>GACCGTACACCGACAAACCT	Core structure	190
**SUR1**	Forward primer<br>Reverse primer	CCTGTATCGAGGCCGAAGAC<br>ATGTCGTCCGGCGTTAGTTT	Core structure	151
**GSTF10**	Forward primer<br>Reverse primer	CTCATGGGCTTTCCTGCTGA<br>AGGGAAGGTGAGCCAAATCG	Core structure	148
**GGP1**	Forward primer<br>Reverse primer	GACGAAGTGTTGGTGTTGCC<br>CTTTGCCGCATCAGCCATTT	Core structure	198
**CYP79B2**	Forward primer<br>Reverse primer	CATCGGAATGGTCCCAACCGA<br>CTCACGCATGCTATCTCGGGT	Core structure	96
**CYP79B3**	Forward primer<br>Reverse primer	TTCGGATCTCACGACCAAGC<br>GAGAGGGTGCAGCTTCTTGT	Core structure	151
**CYP83B1**	Forward primer<br>Reverse primer	TGCAGTGATCTCATCGGCCTG<br>ACGAGCGGTGAAATTGAGGT	Core structure	170
**CYP81F2**	Forward primer<br>Reverse primer	CTGCAGCCGTGACACTAGAA<br>CAACCGGAAGGTCTCGGAAA	Secondary modifications	173
**MYB51**	Forward primer<br>Reverse primer	CACCGTGTTGCAAAGCTGAA<br>GTCCAGCTTTTTCGGGGAGA	Route regulator	129
**ATR1**	Forward primer<br>Reverse primer	CGGACGAACAGACAACGAGA<br>TTGCAACGCGGTTAAGAAGC	Route regulator	182
**GSL-ALK**	Forward primer<br>Reverse primer	ACGAATTACCGTTTGCGACTG<br>ACGTCACCCTTGGCATTAGC	Secondary modifications	188
**GADPH**	Forward primer<br>Reverse primer	TCAGTTGTTGACCTCACGGTT<br>CTGTCACCAACGAAGTCAGT	House Keeping gene	100
